# Washed preparation of faecal microbiota changes the transplantation related safety, quantitative method and delivery

**DOI:** 10.1111/1751-7915.14074

**Published:** 2022-05-16

**Authors:** Gaochen Lu, Weihong Wang, Pan Li, Quan Wen, Bota Cui, Faming Zhang

**Affiliations:** ^1^ Medical Center for Digestive Diseases The Second Affiliated Hospital of Nanjing Medical University Nanjing 210011 China; ^2^ 12461 Key Lab of Holistic Integrative Enterology Nanjing Medical University Nanjing 210011 China; ^3^ Department of Microbiotherapy Sir Run Run Hospital Nanjing Medical University Nanjing 211166 China; ^4^ National Clinical Research Center for Digestive Diseases Xi'an 710032 China

## Abstract

The safety, quantitative method and delivery of faecal microbiota transplantation (FMT) vary a lot from different countries in practice. Recently, the improved methodology of FMT based on the automatic filtration, washing process and the related delivery was named as washed microbiota transplantation (WMT). First, this study aimed to describe the methodology development of FMT from manual to washing preparation from 2012 to 2021 in China Microbiota Transplantation System (CMTS), a centralized stool bank for providing a national non‐profit service. The secondary aim is to describe donor screenings, the correlation between faecal weight and treatment doses, incidence of adverse events and delivery decision. The retrospective analysis on the prospectively recorded data was performed. Results showed that the success rate of donor screening was 3.1% (32/1036). The incidence rate of fever decreased significantly from 19.4% (6/31) in manual FMT to 2.7% (24/902) in WMT in patients with ulcerative colitis (UC), which made UC a considerable disease model to reflect the quality control of faecal microbiota preparation. We defined one treatment unit as 10 cm^3^ microbiota precipitation (1.0 × 10^13^ bacteria) based on enriched microbiota instead of rough faecal weight. For delivering microbiota, colonic transendoscopic enteral tube is a promising way especially for multiple WMTs or frequent colonic administration of drugs combined with WMT. This study should help improve the better practice of FMT for helping more patients in the future.

## Graphical abstract

The safety, quantitative method and delivery of faecal microbiota transplantation (FMT) vary a lot from different countries in practice. Recently, the improved methodology of FMT based on the automatic filtration, washing process and the related delivery was named as washed microbiota transplantation (WMT). The methodology development of FMT from manual to washing preparation was described from 2012 to 2021 in China Microbiota Transplantation System (CMTS), a centralized stool bank for providing a national non‐profit service. This study should help improve the better practice of FMT for helping more patients in the future.

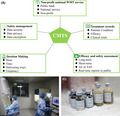

## Introduction

Fecal microbiota transplantation (FMT) has been used in clinical medicine for over one thousand years (Zhang *et al*., 2012). As an effective method for reconstructing the gut microbiota of recipients, FMT is gaining great attention in the increasing clinical research settings, including gastrointestinal disorders, neurological disorders, cardiovascular disease and even cancer (Bajaj *et al*., 2017; Kang *et al*., 2017; Wang *et al*., 2018; Costello *et al*., 2019; Ianiro *et al*., 2020a; Baruch *et al*., 2021). Stool banks are emerging as high‐level facilities to improve the safety and efficacy of FMT.

The recent surveys on doctors, medical students and patients showed that they have a negative perception of FMT (Paramsothy *et al*., 2015a; Ren *et al*., 2016; McSweeney *et al*., 2020), especially due to its manual preparation methods (Zipursky *et al*., 2012, 2014; Wu *et al*., 2019). In 2019, two serious adverse events (SAEs) (death and infection) occurred due to drug‐resistant *E. coli* bacteraemia transmitted by FMT (DeFilipp *et al*., 2019), which aroused the public attention to the safety of FMT. Therefore, physicians seemed to be more receptive to but cautious of FMT with consistently increasing evidence of the safety and efficacy of FMT.

The methodology of FMT based on the automatic washing process and the related delivery since 2014 was named as washed microbiota transplantation (WMT) (Zhang *et al*., 2020a), which was released in Nanjing consensus by the (Fecal Microbiota Transplantation‐standardization Study Group, 2020). The integrated clinical findings, animal experiments and *in vitro* tests demonstrated that the core of WMT is ‘washing’, which significantly decreased adverse events (AEs) in patients with mucosal barrier injury by removing certain bacterial fragments, pro‐inflammatory metabolites, soluble molecules and virus (Zhang *et al*., 2020a). The traditional manual FMT for determining the treatment dose was mainly based on the faecal weight or the volume of faecal water. Washing process can quantify enriched microbiota for precise treatment. Our recent studies indicated that the improved methodology of the washing process does not decrease the efficacy of FMT while improving the safety (Wang *et al*., 2018; Ding *et al*., 2019; Zhang *et al*., 2020a).

We started the stool bank in Nanjing Medical University for clinical research in 2012 (Zhang *et al*., 2013; Cui *et al*., 2015). Then, the hospitalized stool bank was supported by China National Clinical Research Centre for Digestive Diseases in 2015, and was named as fmtBank, a stool bank providing national non‐profit FMT service for refractory intestinal infections (Cui *et al*., 2016; Zhang *et al*., 2018). In 2017, this system was developed as China Microbiota Transplantation System (CMTS). This article aims to report the methodological development of FMT for improving quantitative method, the safety, delivery and the related clinical decision.

## Results

### Donor screening and population recruited

**Fig. 1:**
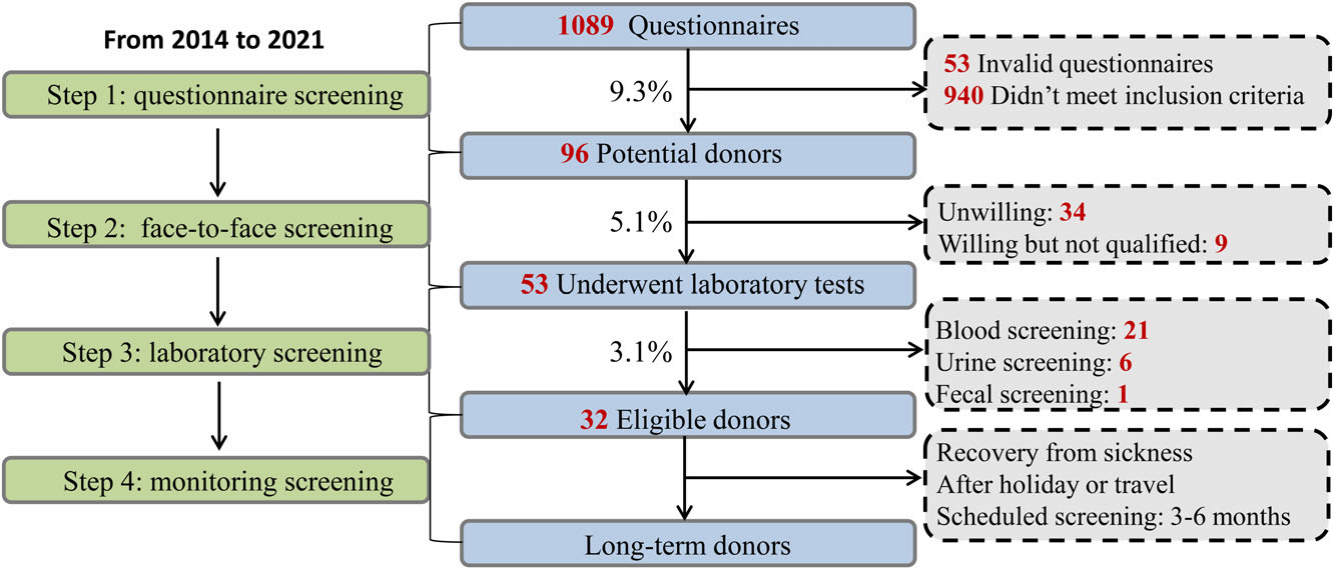
Donor screening flow.

From 2014 to 2021, 1,036 (95.1%, 1036/1089) valid questionnaires were collected from 1,089 individuals (aged 20.54 ± 1.51, BMI 21.04 ± 3.37, Fig. 1). Four hundred and ninety‐five candidates (47.8%, 495/1036) were excluded because of irregular bowel habits and 262 candidates (25.3%, 262/1036) were unqualified due to BMI > 24 or < 18. Among 541 candidates who expressed having regular bowel habits in the questionnaire, there were other reasons for which candidates were excluded. The most common reason for elimination was candidates with abnormal immunity‐related diseases (71.9%, 389/541), followed by typical digestive system diseases (59.3%, 321/541), medication history within 6 months (39.4%, 213/541), family history and genetic diseases (28.1%, 152/541), and infectious diseases (17.2%, 93/541). Thirty‐two eligible donors (3.1%, 32/1036) were finally screened for providing faecal microbiota.

### Fecal weight and treatment

During medical practice, the centre defined one treatment unit (1 U) as 10 cm^3^ microbiota precipitation (1.0 × 10^13^ bacteria) for convenience to calculate the volume of enriched washed microbiota (Group., 2020; Zhang *et al*., 2020a). Generally, a full dose of 5 units is required in single treatment for adults and children over than 7 years old. And the dose of enriched microbiota for children from 1–7 year‐old ranged from 1 to 5 units (Group., 2020; Zhang *et al*., 2020a). The frequency of WMTs for most patients in each hospitalization case ranges from one to three times.

**Fig. 2:**
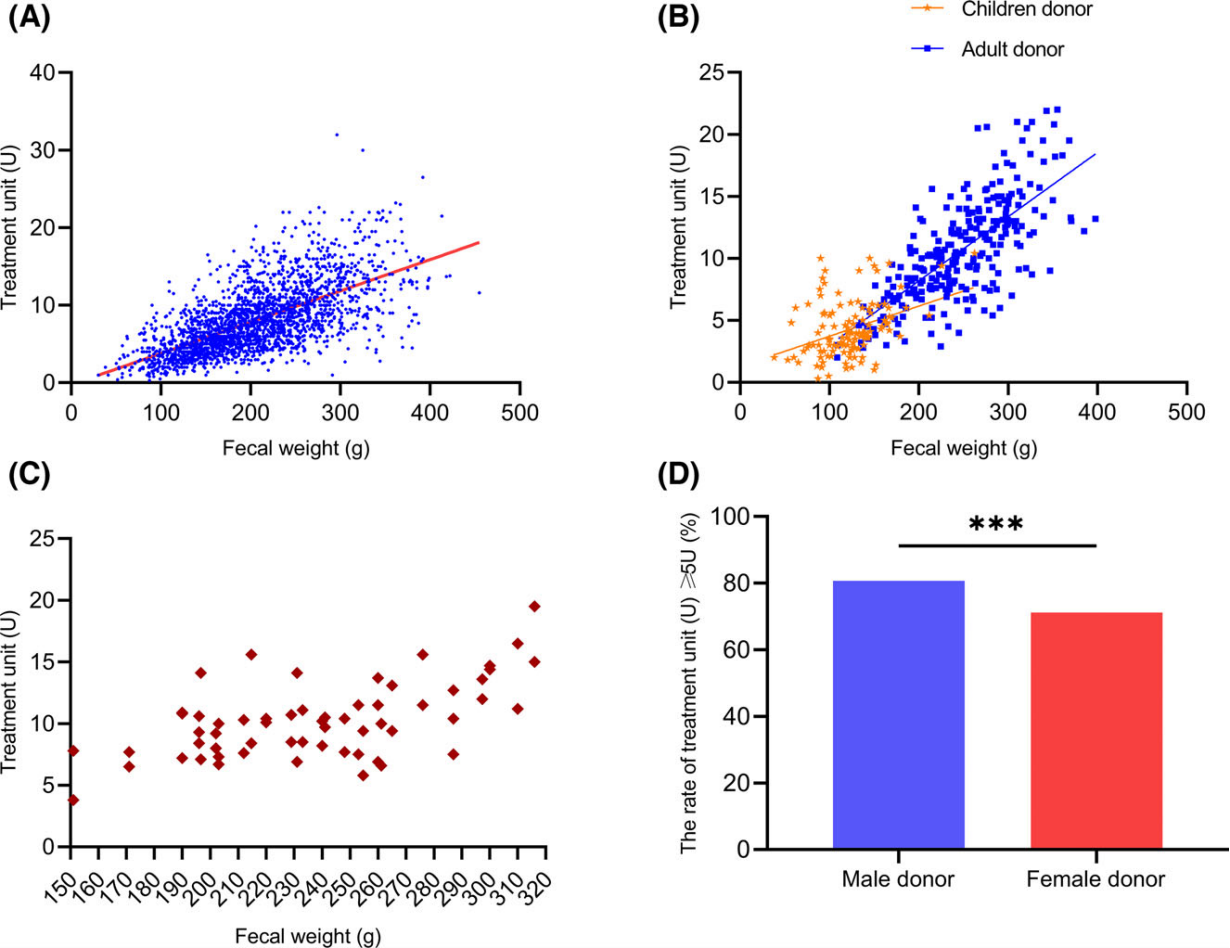
Correlation between the faecal weight and the amount of enriched washed microbiota. A. Correlation between the faecal weight and enriched microbiota in WMT donors (*n* = 2517). B. Difference in correlation between the fecal weight and enriched microbiota from one children donor (*n* = 124) and one adult donor (*n* = 247). C. Different doses of enriched microbiota among the same weight of faeces. D. The rate of treatment unit differs between male and female donor. Correlation analysis was performed using Spearman correlation analysis. Statistical comparisons were performed using chi‐square test; ^*^ *P* < 0.05, ^**^ *P* < 0.01, ^***^ *P* < 0.001.

As shown in Fig. 2A, among total 2517 donations, the faecal weight was not well correlated with the amount of enriched washed microbiota (which we defined as treatment unit) (*r* = 0.65, 95% CI, 0.63–0.68, *P* < 0.0001), the correlation between faecal weight and the amount of enriched washed microbiota differs from each other in children donor (*r* = 0.37, 95% CI, 0.21–0.52, *P* < 0.0001, Fig. 2B) and adult donor (*r* = 0.71, 95% CI, 0.64–0.76, *P* < 0.0001, Fig. 2B). Even for the same weight of faeces from different defecation samples in one donor, the relationship between the faecal weight and enriched microbiota was not satisfactory (Fig. 2C).

**Fig. 3:**
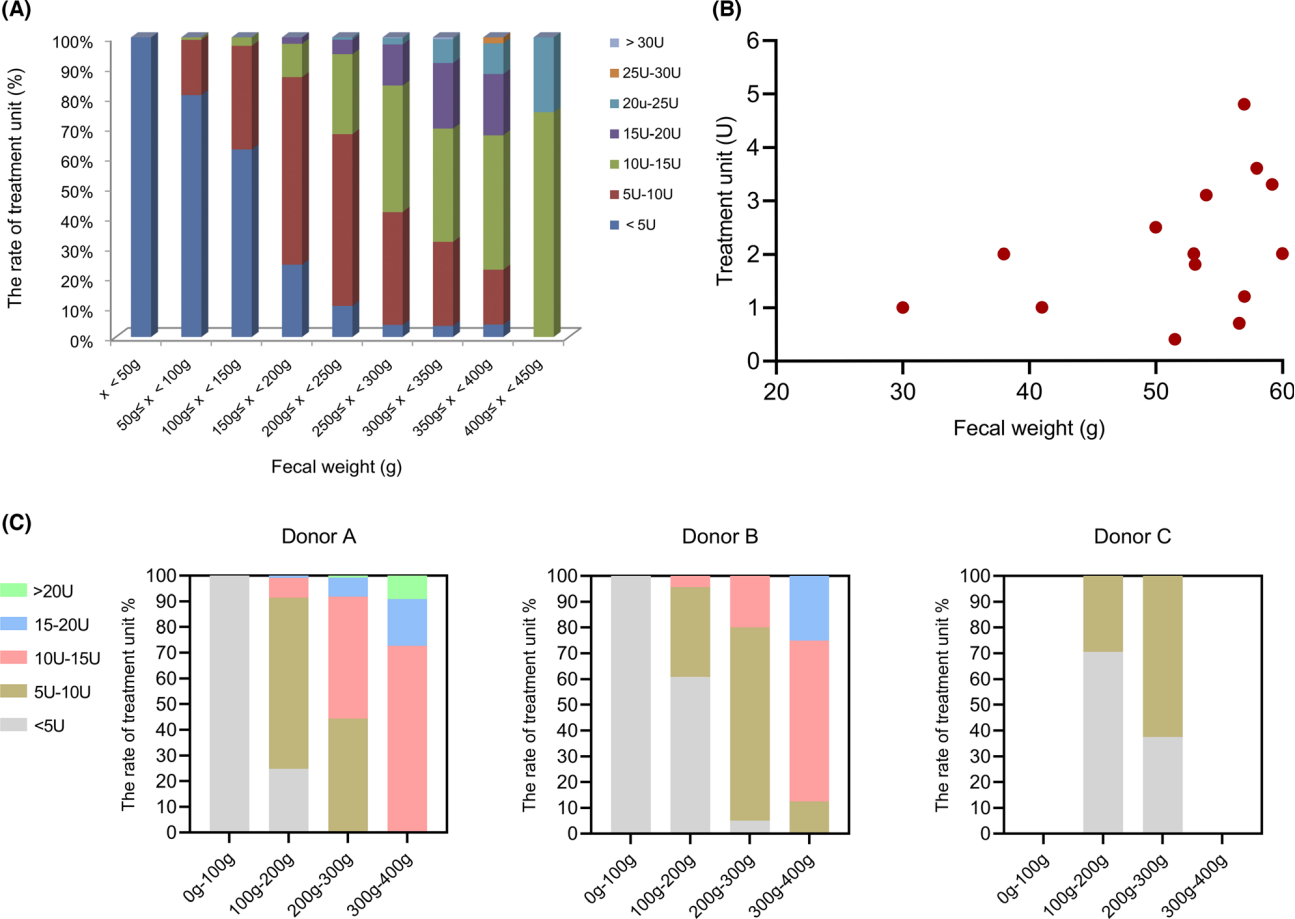
Specific distribution of faecal weight and the related treatment unit. A. *X*‐axis: The distribution of faecal weight. *Y*‐axis: The rate of treatment unit. B. The detailed distribution of treatment unit among faecal weight less than 60 g. C. Different distribution of treatment unit among three different donors.

Generally, the amount of enriched microbiota accumulates with the increase of faecal weight. Based on the stratified analysis of faecal weight, donations that meet a minimum weight of 150 g are cost‐effective to handle because more than 75% (525/692) of which can be used to treat at least one patient (Fig. 3A). Donations less than 50 g cannot even meet the basic treatment unit for single WMT (Fig. 3B).

Even for the same range of faecal weight, the microbiota output differs from each other among different donors. As shown in Fig. 3C, donor A showed more doses of microbiota output than donor B and C among each range of faecal weight. Male donors are more likely to have better microbiota output than female donors (*P* < 0.001) (Fig. 2D).

### Different delivering ways

**Fig. 4:**
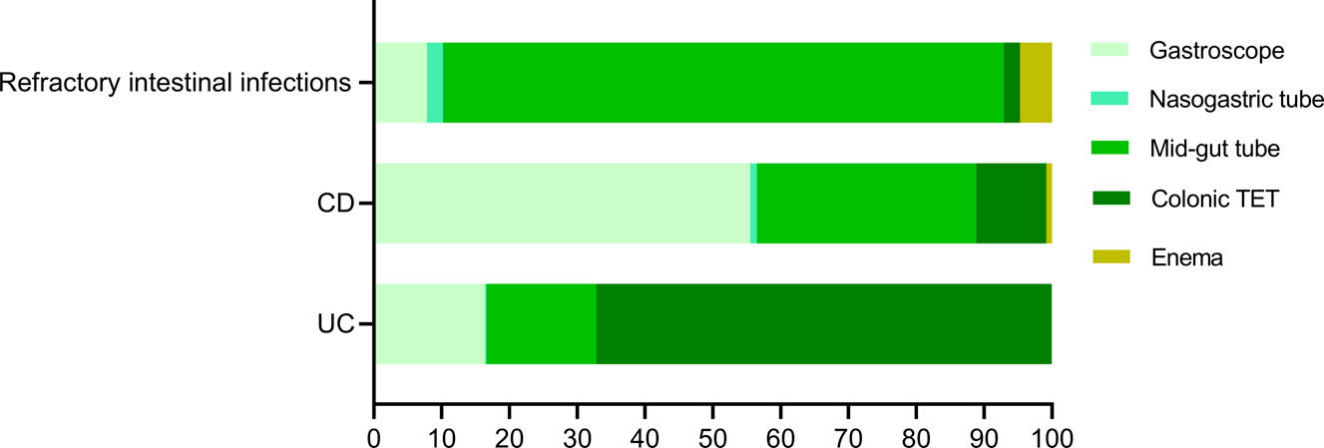
Different delivering ways for WMT in patients with UC, CD and refractory intestinal infections. The *X*‐axis shows the percentage of different delivering ways. CD, Crohn’s disease; TET, transendoscopic enteral tube; UC, ulcerative colitis.

Colonic transendoscopic enteral tube (TET) is recommended for patients who need multiple WMTs or colonic administration of drugs combined with WMT. Figure 4 showed that delivering WMT through colonic TET is a primary method for ulcerative colitis (UC) (67.2%, 627/933). While gastroscope (55.5%, 528/951) and mid‐gut tube (32.4%, 308/951) are two predominant mid‐gut delivering ways in patients with Crohn’s disease (CD). For refractory intestinal infections rescued by WMT, 82.8% (211/255) of patients underwent WMT through mid‐gut tube. No tube obstruction was observed through mid‐gut or colonic TET for WMT.

### Microbiota transplantation ‐related adverse events

**Fig. 5:**
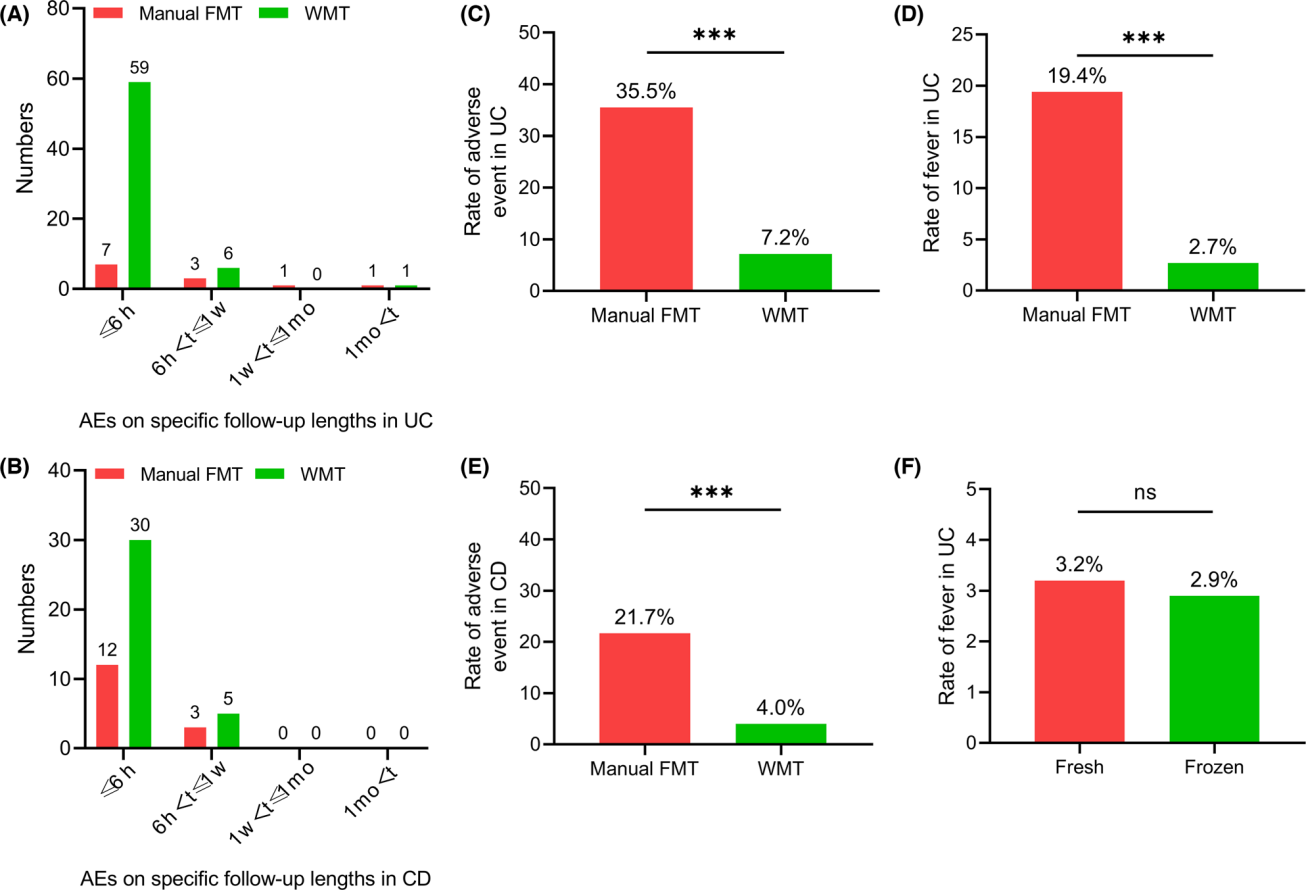
The incidence rate of microbiota transplantation related adverse events. A–B. The frequency numbers of AEs on specific follow‐up lengths related to manual FMT and WMT in patients with UC and CD respectively. C. The rate of short‐term AEs related to manual FMT and WMT in patients with UC. D. The rate of fever related to manual FMT and WMT in short‐term follow‐up in patients with UC. E. The rate of short‐term AEs related to manual FMT and WMT in patients with CD. F. The rate of fever related to fresh and frozen microbiota in short‐term follow‐up in patients with UC. AE, adverse events; CD, Crohn’s disease; FMT, fecal microbiota transplantation; *t*, time; UC, ulcerative colitis; WMT, washed microbiota transplantation. Statistical comparisons were performed using chi‐square test; ns: no significance, *P < 0.05, **P < 0.01, ***P < 0.001.

The current data from a total of 574 patients with inflammatory bowel disease (285 UC and 289 CD) who underwent FMT/WMT were recorded in CMTS from 2012 to 2021. These patients were classified as those who had mucosal barrier injury. When using 1 month cut‐off to define short‐term or long‐term AEs related to FMT/WMT, analysis showed that only two AEs were reported to occur one month after treatment, which was consistent with previous findings (Fig. 5A and B). During short‐term follow‐up, the rate of [Bibr mbt214074-bib-0064] AEs in patients with UC significantly decreased from 35.5% (11/31) by manual FMT to 7.2% (65/902) by WMT (*P* < 0.001, Fig. 5C). The rate of AEs in patients with CD decreased from 21.7% (15/69) to 4% (35/882) (*P* < 0.001, Fig. 5E).

The rate of fever after microbiota transplantation significantly decreased from 19.4% (6/31) in manual FMT to 2.7% (24/902) in WMT in patients with UC (*P* < 0.001, Fig. 5D). However, the rate of fever was not related to the fresh or frozen status of microbiota storage in patients with UC (3.2% vs. 2.9%, 28/863 and 2/70 respectively, *P* > 0.05, Fig. 5F).

## Discussion

The methodological development from FMT to WMT mainly involves donor screening, laboratory preparation and delivery. In CMTS, the candidate donors are mainly from college students and children and the success rate of donor screening was 3.1%. The rate varies from different stool banks, such as OpenBiome (3%) in the United States (Kassam *et al*., 2019), Canada (2.2%) (Craven *et al*., 2017), the Netherlands (2.4%) (Terveer *et al*., 2017), Australian (10%) (Paramsothy *et al*., 2015b) and Italy (25%) (Ianiro *et al*., 2021). Importantly, the methods on donor screening were changing dynamically all over the world (Woodworth *et al*., 2017; Ianiro *et al*., 2020b; McSweeney *et al*., 2020).

We found that the faecal weight was not well correlated with the amount of enriched microbiota. Quantified washed microbiota preparations can provide precise doses of microbiota. The analysis on the precise dose of enriched faecal microbiota showed that the faecal weight less than 50 g was not cost‐effective and provided very few doses for WMT treatment, thus faeces from donors less than 50 g were not recommended in Nanjing consensus. We defined 10 cm^3^ microbiota precipitation (1.0 × 10^13^ microbial cells) as one basic treatment unit (Group., 2020; Zhang *et al*., 2020b). Our treatment strategy is 1–5 units for each delivery in children and adults. The present findings highlight the significance of metrological faecal microbiota and a favourable cost‐effectiveness during the washing process.

The delivery decision is made based on the clinician's judgement of patients’ condition. For example, patients who have difficulty in swallowing capsules may choose mid‐gut tubes (nasojejunal tube, gastrostomy tube or jejunostomy tube). Mid‐gut tube is useful for repeated infusion of microbiota and the combination of enteral nutrition. As for patients who are not suitable for the upper gastrointestinal delivery, lower gastrointestinal delivery such as colonic TET, colonoscopy and enema can be taken into consideration. Since the treatment dose is based on washed microbial cells, different delivering ways have no effect on it. However, different studies which use manual faecal microbiota suspension recommend different treatment volume of suspension according to upper or lower gastrointestinal delivery because of physiological structure (different parts of the digestive tract can hold different volumes of fluid). The data from this real‐world study indicated that colonic TET is the predominant delivery for WMT. The safety of colonic TET has already been proved in adults (≥ 18 years old) and children aged over 3 years old (Zhang *et al*., 2020b; Zhong *et al*., 2021a, 2021b,2021a, 2021b). Colonic TET is helpful to increase the frequency of WMT for increasing the efficacy, which is the aim of step‐up WMT strategy (Ding *et al*., 2020; Xiang *et al*., 2020). A prospective study including 224 patients reported 97.8% of satisfaction with the colonic TET, and the success rate of colon TET was 100% (Zhang *et al*., 2020b). Recently, the colonic TET was first time used to rescue endoscopy‐associated perforation, increasing our confidence in the different usage of colonic TET (Zhang *et al*., 2021a, 2021b,2021a, 2021b). Colonic TET is also a convenient way for local microbiota analysis because researchers could directly acquire microbiota through this tube (Liu *et al*., 2021). However, the colonic TET is not recommended for delivering the manual preparation of FMT because of the possible obstruction in tube (Wang *et al*., 2019).

This study further confirms our previous finding that washing preparation is an independent contributor to reduce the incidence rate of FMT‐related AEs by improving intestinal mucosal permeability and decreasing pro‐inflammatory metabolites (Zhang *et al*., 2020a). So far, water, sterile saline, phosphate‐buffered saline have been used as a vector solvent of faecal material (Liao and Shollenberger, 2003; Mattila *et al*., 2012; Cammarota *et al*., 2017, 2019). Normal saline is used to prepare most faecal microbiota suspensions, as this solvent enables better preservation of microbes (Mattila *et al*., 2012). Metabolism analysis has proved a significant decrease in pro‐inflammatory metabolites during the washing process such as prostaglandin G2, leukotriene B4, TRPV1 and the related differentially enriched metabolic pathways, which play important roles in fever and inflammation. Metagenomic next‐generation sequencing (NGS) indicated the increasing types and amount of viruses could be washed out during the washing process. Further animal experiments and *in vitro* screening also supported the evidence for linking the clinical findings to the safety of WMT (Zhang *et al*., 2020a). Published data from CMTS have shown the reliable safety of rescue WMT in critically ill patients (Dai *et al*., 2019) and patients with graft‐versus‐host disease (Qi *et al*., 2018). The recent studies in other centres showed the similar findings on the safety and efficacy of WMT (Chen *et al*., 2020; Zheng *et al*., 2021; Zhong *et al*., 2021a, 2021b,2021a, 2021b).

AEs were generally calculated in two ways: the frequency of AEs post‐FMT during follow‐up, or the proportion of patients with symptoms. Recently, Kelly *et al*. reported the initial results from the American Gastroenterological Association FMT National Registry programme using the proportion of patients with symptoms to evaluate the safety of FMT in CDI (Kelly *et al*., 2021). Mayo Clinic reported the incidence rate of AEs calculated by the proportion of patients with symptoms: 13% gastrointestinal symptoms, 10% weight gain and 11.8% new infections (Saha *et al*., 2021). In our recent systematic review, the global data calculated by using the frequency of AEs post‐FMT during follow‐up showed that the incidence rate of FMT‐related AEs, FMT‐related SAEs and death was observed in 19%, 1.39% and 0.12% of total FMT courses, respectively (Marcella *et al*., 2021), which shows a higher trend than the online data of CMTS (http://fmtbank.org/).

Which item should be selected as a quality control model of WMT is another practical issue. According to this study, since one month post‐FMT was regarded as a cut‐off between short‐term and long‐term follow‐up, more attention needs to be paid to the short‐term evaluation. Our recent systematic review on the global incidence of FMT‐related AEs from 2000 to 2020 demonstrated that FMT‐related SAEs, including serious infections and deaths, had been reported in 1.4% of patients who underwent FMT (0.99% microbiota‐related SAEs) (Marcella *et al*., 2021). All reported FMT‐related SAEs were happened in patients with mucosal barrier injury, which was defined as endoscopically confirmed mucosa broken (e.g. ulcerative or erosive lesions) or clinically confirmed acute intestinal infections with determined or undetermined pathogens (See *et al*., 2013; Marcella *et al*., 2021). Based on our previous study, Wang *et al*. reported a 1 month cut‐off could be suggested to define short‐term and long‐term AEs of FMT/WMT because no AE beyond 1 month was observed in a total of 184 frequencies of FMT/WMT in CD patients. During a follow‐up of up to eight years, only two patients reported myasthenia gravis and rash/pruritus, respectively 1 month after FMT/WMT. The remaining AEs all happened within 1 month, especially within 6 hours after FMT/WMT. Therefore, the incidence rate of fever as a short‐term FMT/WMT‐related AE in patients with UC should be considered as a valuable indicator to evaluate the quality of laboratory processes for WMT. In another words, the safety of microbiota transplantation in patients with light intestinal barrier injury or even without mucosa damage, such as constipation, irritable bowel syndrome and autism, cannot really reflect the quality control of delivered microbiota.

The development of WMT from FMT is similar to the history of blood transfusion. The revolutionary development of blood transfusion significantly improves the safety (Goodnough *et al*., 2013). This public platform works hard to promote the transformation of WMT from clinical benefits to breakthrough in disease cognition. The latest pilot studies indicate the role of WMT on radiation colitis (Ding *et al*., 2020), complex infections (Dai *et al*., 2019), recurrent invasive fungal infection (Wu *et al*., 2021), refractory *Helicobacter pylori* infection (Ye *et al*., 2020).

However, there are limitations in this study. We did not integrate the microbiome analysis and efficacy evaluation on WMT, although we have reported these in our previous studies. The policy on the use of FMT in China is the permitted medical therapy for CDI and many other diseases. This is the reason why we focused on the studies in populations beyond CDI, which has already been well studied in Europe and North America (Allegretti *et al*., 2019; Baunwall *et al*., 2021).

In conclusion, donor screening, washing process, dose, time, frequency and delivery were all closely related to the safety and efficacy of WMT. The experience of washed preparation of faecal microbiota should be helpful to improve the better practice of FMT for helping more patients in the future.

## Experimental procedures

### Microbiota transplantation system and data management

**Fig. 6:**
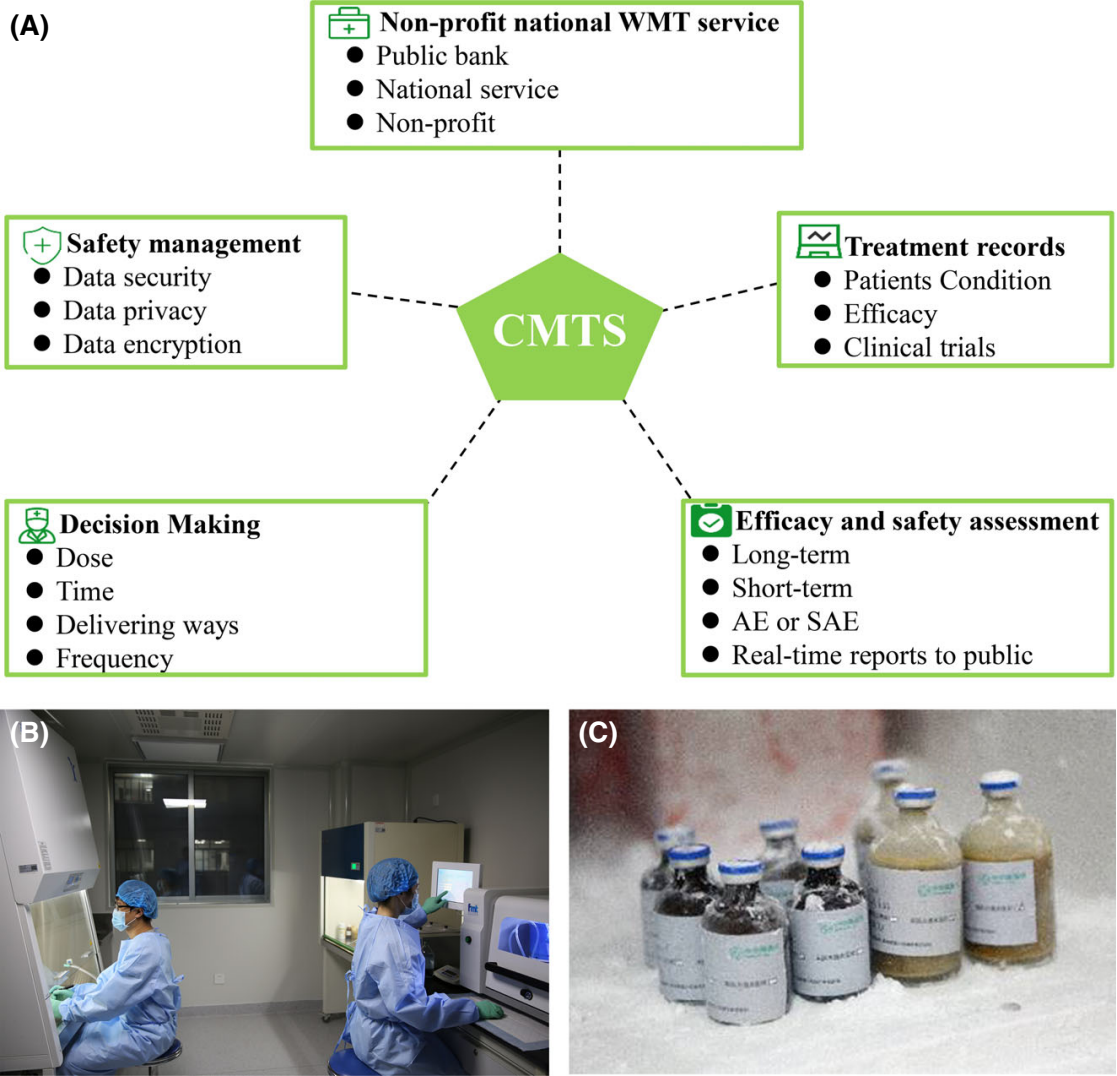
The framework and laboratory of CMTS. A. The function framework of CMTS. B. The laboratory for washed microbiota preparation. C. The frozen washed microbiota. AE, adverse event; CMTS, China Microbiota Transplantation System; SAE, serious adverse event; WMT, washed microbiota transplantation.

CMTS aims to support non‐profit WMT practice and research in China for long‐term evaluation of the decision, treatment, efficacy and safety of microbiota transplant (Fig. 6A). The data were recorded by research teams and doctors.

### Donor recruitment and evaluation

Faeces donations were approved by the institutional ethics committee of the Second Affiliated Hospital of Nanjing Medical University. Healthy adults and adolescents (preferably aged 6–24 years old) are potential donors in the clinical practice according to the consensus (Group., 2020). Candidates were informed of the potential risks and benefits of WMT for recipients (Wu *et al*., 2019; McSweeney *et al*., 2020) and should provide written informed consent. Children donors should be screened after obtaining parental consent. Questionnaire screening, face‐to‐face screening and laboratory screening are taken step‐by‐step to exclude candidates based on the criteria including age, physiology, pathology, psychology, integrity, time, environment and recipient status (Ding *et al*., 2019; Zhang *et al*., 2019).

### Washed microbiota preparation and quality control

The method for preparation of microbiota is based on the automatic microbiota purification system (GenFMTer, FMT Medical, Nanjing, China) followed with centrifugation plus suspension three times in a specially designed exclusive laboratory at good manufacture practice level (Cui *et al*., 2016; Zhang *et al*., 2018) (Fig. 6B). The time from defecation of a donor, laboratory preparation for enriching microbiota to the time of microbiota delivering or microbiota storing was limited within one hour (He *et al*., 2017; Zhang *et al*., 2018; Ding *et al*., 2019), which was called ‘one‐hour protocol’ (Group., 2020).

Faeces are collected on‐site by a disposable faeces container in a dedicated room only for donors. The donated faeces (generally ≥ 50 g) and sterile saline were put together into the GenFMTer at the ratio of 500 ml of saline per 100 g faeces to become faecal microbiota suspension. The faecal suspension is transferred to centrifugation tubes for centrifugation with 700 *g* for 3 min and the supernatant is discarded. The above centrifugation plus washing and suspension using sterile saline should be repeated three times (Zhang *et al*., 2020a). Washed microbiota suspension consists of final precipitation and vector solution with the volume ratio of 1:2 for fresh use. Frozen samples can be safely stored by mixed with 10% glycerol at −80°C for frozen use. The frozen samples should be thawed in a warm water bath (37°C water temperature, 30–45 min from −80°C to 37°C). Retention of stool samples is recommended for ‘look‐ back’ testing.

During medical practice, we defined one treatment unit (1 U) as 10 cm^3^ microbiota precipitation (1.0 × 10^13^ bacteria) to calculate the volume of enriched washed microbiota. We used fluorescent staining for total bacteria counts (Magge *et al*., 2009; Emerson *et al*., 2017).

### Delivering ways for washed microbiota transplantation

The delivery of microbiota transplantation includes the upper gut, mid‐gut and lower gut (Peng *et al*., 2016). Oral intake (capsules or drinking) and nasogastric tube are two means of upper gut delivery way (Lee *et al*., 2016; Zhang *et al*., 2021a, 2021b,2021a, 2021b). Mid‐gut delivery includes infusing the microbiota suspension into the small intestine beyond the second duodenal segment through endoscopy and mid‐gut tube (nasojejunal tube or mid‐gut TET) (Cui *et al*., 2015; Long *et al*., 2018). Faecal microbiota can also be delivered to the lower gut through colonoscopy, enema, stoma in ileocolon and colonic TET in adults and paediatrics (Peng *et al*., 2016; Wen *et al*., 2020; Zhong *et al*., 2021a, 2021b,2021a, 2021b). The endoscopic delivery and TET technique can be performed by endoscopist and gastroenterologist in the CMTS.

### Clinical trials and non‐profit national washed microbiota transplantation service

The clinical data of this study was based on our pooled registered trials for UC, CD and refractory intestinal infections (ClinicalTrials.gov, NCT01790061, NCT01793831, and NCT03895593). The system also records the information about the population for attending clinical trials such as autism, epilepsy and radiation enteritis. However, data from these trials were not included in this study. CMTS provides rescue WMT service for patients nationally (Fig. 6C) (Dai *et al*., 2019). The rescue WMT is a teamwork in which there are at least two intensive care specialists in charge from destination hospitals, professional WMT clinicians, laboratory managers and clinical research coordinators from the CMTS. The rescue team members communicated closely throughout the whole process to ensure the safety fluent work flow of WMT. Conditions, contraindications and the potential risk factors of SAEs were evaluated by the team. Rescue WMT has been successfully applied in treating GVHD and antibiotic‐associated diarrhoea (Qi *et al*., 2018; Huang *et al*., 2020; Wang *et al*., 2021).

### Safety evaluation for washed microbiota transplantation

In this study, any new onset of symptom and the exacerbation of previous symptoms were recorded and evaluated according to the Code of Federal Regulations and researchers’ clinical experience. SAEs include: an AE that is disease spreading, fatal, or life threatening, or requires professional intervention that requires hospitalization or prolonged hospital stays, or results in infertility, congenital anomaly, permanent disability or disfigurement. Daily records were used for AEs within one week after treatment. AEs beyond hospital were recorded via telephone follow‐up or hospital visits. AEs were evaluated at 6 h, 1 week, 1 month, 3 months, 6 months, 1 year and every year after FMT/WMT. A one month cut‐off could be suggested to define short‐term and long‐term AEs of FMT/WMT according to previous study (Wang *et al*., 2018). Causality between AEs and FMT/WMT was categorized as definitely related, probably related, possibly related and unrelated (Kelly *et al*., 2014). Only FMT/WMT‐related (including definitely, probably and possibly related) AEs were reported in this study. A group discussion from two or more physicians is needed to form a common opinion once the relativity of AEs could not be identified. The recorded incidence rate of AEs/SAEs was calculated using the frequency of AEs to the total FMT courses ratio.

### Statistical analysis

Descriptive analysis was used in this article. Patient characteristics were evaluated using proportions for categorical variables. Categorical variables were analysed by chi‐square test. Pearson correlation coefficient is used to describe the degree of correlation between two variables. *P* values < 0.05 were considered significant. Data were analysed by IBM SPSS 24.0 or GraphPad 7.0.

### Ethic approval

All procedures followed were in accordance with the ethical standards of the institutional review board of the Second Affiliated Hospital of Nanjing Medical University and with the Helsinki Declaration of 1975, as revised in 2000. The clinical data of this study was based on our pooled registered trials for UC, CD and refractory intestinal infections (ClinicalTrials.gov, NCT01790061, NCT01793831, and NCT03895593). Informed consent prior to participate was obtained from all patients for being included in the study. For the participants aged below 18, informed consent was obtained from their parents/legally authorized representatives.

## Conflicts of interest

FZ conceived the concept of GenFMTer and transendoscopic enteral tubing and devices related to them. The remaining authors declare that they have no competing interests.

## Author contributions

GL and WW designed the study, analysed data and wrote the manuscript. PL, QW and BC participated in the data collection. FZ had the study conception, manuscript revision and grants support.

## Data Availability

The data are not publicly available due to the containing information that could compromise research participants’ privacy/consent. The basic data that support the findings of this study are available on request from the corresponding author’s e‐mail.

## References

[mbt214074-bib-0001] Allegretti, J.R. , Mullish, B.H. , Kelly, C. , and Fischer, M. (2019) The evolution of the use of faecal microbiota transplantation and emerging therapeutic indications. Lancet 394: 420–431. 31379333 10.1016/S0140-6736(19)31266-8

[mbt214074-bib-0002] Bajaj, J.S. , Kassam, Z. , Fagan, A. , Gavis, E.A. , Liu, E. , Cox, I.J. , *et al*. (2017) Fecal microbiota transplant from a rational stool donor improves hepatic encephalopathy: a randomized clinical trial. Hepatology 66: 1727–1738. 28586116 10.1002/hep.29306 PMC6102730

[mbt214074-bib-0064] Baunwall, S.M.D. , Terveer, E.M. , Dahlerup, J.F. , Erikstrup, C. , Arkkila, P. , Vehreschild, M.J. , *et al*. (2021) The use of Faecal Microbiota Transplantation (FMT) in Europe: A Europe‐wide survey. Lancet Reg Health Eur 9: 100181. 34693388 10.1016/j.lanepe.2021.100181 PMC8513118

[mbt214074-bib-0003] Baruch, E.N. , Youngster, I. , Ben‐Betzalel, G. , Ortenberg, R. , Lahat, A. , Katz, L. , *et al*. (2021) Fecal microbiota transplant promotes response in immunotherapy‐refractory melanoma patients. Science 371: 602–609. 33303685 10.1126/science.abb5920

[mbt214074-bib-0004] Cammarota, G. , Ianiro, G. , Tilg, H. , Rajilić‐Stojanović, M. , Kump, P. , Satokari, R. , *et al*. (2017) European consensus conference on faecal microbiota transplantation in clinical practice. Gut 66: 569–580. 28087657 10.1136/gutjnl-2016-313017 PMC5529972

[mbt214074-bib-0005] Cammarota, G. , Ianiro, G. , Kelly, C.R. , Mullish, B.H. , Allegretti, J.R. , Kassam, Z. , *et al*. (2019) International consensus conference on stool banking for faecal microbiota transplantation in clinical practice. Gut 68: 2111–2121. 31563878 10.1136/gutjnl-2019-319548 PMC6872442

[mbt214074-bib-0006] Chen, M. , Liu, X. , Zhang, Y. , Nie, Y. , Wu, K. , and Shi, Y. (2020) Efficacy and safety of fecal microbiota transplantation by washed preparation in patients with moderate to severely active ulcerative colitis. J Dig Dis 21: 621–628. 32909356 10.1111/1751-2980.12938 PMC7756426

[mbt214074-bib-0007] Costello, S.P. , Hughes, P.A. , Waters, O. , Bryant, R.V. , Vincent, A.D. , Blatchford, P. , *et al*. (2019) Effect of fecal microbiota transplantation on 8‐week remission in patients with ulcerative colitis: a randomized clinical trial. JAMA 321: 156–164. 30644982 10.1001/jama.2018.20046 PMC6439766

[mbt214074-bib-0008] Craven, L. , Nair Parvathy, S. , Tat‐Ko, J. , Burton, J. , and Silverman, M. (2017) Extended screening costs associated with selecting donors for fecal microbiota transplantation for treatment of metabolic syndrome‐associated diseases. Open Forum Infect Dis 4: ofx243. 29255739 10.1093/ofid/ofx243 PMC5730934

[mbt214074-bib-0009] Cui, B. , Feng, Q. , Wang, H. , Wang, M. , Peng, Z. , Li, P. , *et al*. (2015) Fecal microbiota transplantation through mid‐gut for refractory Crohn's disease: safety, feasibility, and efficacy trial results. J Gastroenterol Hepatol 30: 51–58. 25168749 10.1111/jgh.12727

[mbt214074-bib-0010] Cui, B. , Li, P. , Xu, L. , Peng, Z. , Xiang, J. , He, Z. , *et al*. (2016) Step‐up fecal microbiota transplantation (FMT) strategy. Gut Microbes 7: 323–328. 26939622 10.1080/19490976.2016.1151608 PMC4988439

[mbt214074-bib-0011] Dai, M. , Liu, Y. , Chen, W. , Buch, H. , Shan, Y.I. , Chang, L. , *et al*. (2019) Rescue fecal microbiota transplantation for antibiotic‐associated diarrhea in critically ill patients. Crit Care 23: 324. 31639033 10.1186/s13054-019-2604-5 PMC6805332

[mbt214074-bib-0012] DeFilipp, Z. , Bloom, P.P. , Torres Soto, M. , Mansour, M.K. , Sater, M.R.A. , Huntley, M.H. , *et al*. (2019) Drug‐resistant E. coli bacteremia transmitted by fecal microbiota transplant. N Engl J Med 381: 2043–2050. 31665575 10.1056/NEJMoa1910437

[mbt214074-bib-0013] Ding, X. , Li, Q. , Li, P. , Zhang, T. , Cui, B. , Ji, G. , *et al*. (2019) Long‐term safety and efficacy of fecal microbiota transplant in active ulcerative colitis. Drug Saf 42: 869–880. 30972640 10.1007/s40264-019-00809-2

[mbt214074-bib-0014] Ding, X. , Li, Q. , Li, P. , Chen, X. , Xiang, L. , Bi, L. , *et al*. (2020) Fecal microbiota transplantation: a promising treatment for radiation enteritis? Radiother Oncol 143: 12–18. 32044171 10.1016/j.radonc.2020.01.011

[mbt214074-bib-0015] Emerson, J.B. , Adams, R.I. , Roman, C.M.B. , Brooks, B. , Coil, D.A. , Dahlhausen, K. , *et al*. (2017) Schrodinger's microbes: tools for distinguishing the living from the dead in microbial ecosystems. Microbiome 5: 86. 28810907 10.1186/s40168-017-0285-3 PMC5558654

[mbt214074-bib-0017] Fecal Microbiota Transplantation‐standardization Study Group . (2020) Nanjing consensus on methodology of washed microbiota transplantation. Chin Med J (Engl) 133: 2330–2332. 32701590 10.1097/CM9.0000000000000954 PMC7546843

[mbt214074-bib-0016] Goodnough, L. , Levy, J. , and Murphy, M. (2013) Concepts of blood transfusion in adults. Lancet 381: 1845–1854. 23706801 10.1016/S0140-6736(13)60650-9

[mbt214074-bib-0018] He, Z. , Li, P. , Zhu, J. , Cui, B. , Xu, L. , Xiang, J. , *et al*. (2017) Multiple fresh fecal microbiota transplants induces and maintains clinical remission in Crohn's disease complicated with inflammatory mass. Sci Rep 7: 4753. 28684845 10.1038/s41598-017-04984-z PMC5500501

[mbt214074-bib-0019] Huang, G.Q. , Bai, Y. , Sun, Z.Q. , and Liu, J. (2020) Successful treatment of pseudomembranous colitis with fecal microbiota transplantation – a case study on a patient rescued by extracorporeal cardiopulmonary resuscitation after cardiac arrest. Ann Transplant 25: e923283. 32657282 10.12659/AOT.923283 PMC7353294

[mbt214074-bib-0020] Ianiro, G. , Rossi, E. , Thomas, A.M. , Schinzari, G. , Masucci, L. , Quaranta, G. , *et al*. (2020a) Faecal microbiota transplantation for the treatment of diarrhoea induced by tyrosine‐kinase inhibitors in patients with metastatic renal cell carcinoma. Nat Commun 11: 4333. 32859933 10.1038/s41467-020-18127-y PMC7455693

[mbt214074-bib-0021] Ianiro, G. , Mullish, B.H. , Kelly, C.R. , Kassam, Z. , Kuijper, E.J. , Ng, S.C. , *et al*. (2020b) Reorganisation of faecal microbiota transplant services during the COVID‐19 pandemic. Gut 69: 1555–1563. 32620549 10.1136/gutjnl-2020-321829 PMC7456726

[mbt214074-bib-0022] Ianiro, G. , Porcari, S. , Bibbò, S. , Giambò, F. , Quaranta, G. , Masucci, L. , *et al*. (2021) Donor program for fecal microbiota transplantation: a 3‐year experience of a large‐volume Italian stool bank. Dig Liver Dis 53: 1428–1432. 34030988 10.1016/j.dld.2021.04.009

[mbt214074-bib-0023] Kang, D.‐W. , Adams, J.B. , Gregory, A.C. , Borody, T. , Chittick, L. , Fasano, A. , *et al*. (2017) Microbiota Transfer Therapy alters gut ecosystem and improves gastrointestinal and autism symptoms: an open‐label study. Microbiome 5: 10. 28122648 10.1186/s40168-016-0225-7 PMC5264285

[mbt214074-bib-0024] Kassam, Z. , Dubois, N. , Ramakrishna, B. , Ling, K. , Qazi, T. , Smith, M. , *et al*. (2019) Donor screening for fecal microbiota transplantation. N Engl J Med 381: 2070–2072. 31665572 10.1056/NEJMc1913670

[mbt214074-bib-0025] Kelly, C.R. , Kunde, S.S. , and Khoruts, A. (2014) Guidance on preparing an investigational new drug application for fecal microbiota transplantation studies. Clin Gastroenterol Hepatol 12: 283–288. 24107393 10.1016/j.cgh.2013.09.060 PMC3947095

[mbt214074-bib-0026] Kelly, C.R. , Yen, E.F. , Grinspan, A.M. , Kahn, S.A. , Atreja, A. , Lewis, J.D. , *et al*. (2021) Fecal microbiota transplantation is highly effective in real‐world practice: initial results from the FMT National Registry. Gastroenterology 160: 183–192.e3. 33011173 10.1053/j.gastro.2020.09.038 PMC8034505

[mbt214074-bib-0027] Lee, C.H. , Steiner, T. , Petrof, E.O. , Smieja, M. , Roscoe, D. , Nematallah, A. , *et al*. (2016) Frozen vs fresh fecal microbiota transplantation and clinical resolution of diarrhea in patients with recurrent clostridium difficile infection: a randomized clinical trial. JAMA 315: 142–149. 26757463 10.1001/jama.2015.18098

[mbt214074-bib-0028] Liao, C.H. , and Shollenberger, L.M. (2003) Survivability and long‐term preservation of bacteria in water and in phosphate‐buffered saline. Lett Appl Microbiol 37: 45–50. 12803555 10.1046/j.1472-765x.2003.01345.x

[mbt214074-bib-0029] Liu, X. , Dai, M. , Ma, Y. , Zhao, N. , Wang, Z. , Yu, Y. , *et al*. (2021) Reconstruction and dynamics of the human intestinal microbiome observed in situ. Engineering. 10.1016/j.eng.2021.03.015.

[mbt214074-bib-0030] Long, C. , Yu, Y. , Cui, B. , Jagessar, S.A.R. , Zhang, J. , Ji, G. , *et al*. (2018) A novel quick transendoscopic enteral tubing in mid‐gut: technique and training with video. BMC Gastroenterol 18: 37. 29534703 10.1186/s12876-018-0766-2 PMC5850973

[mbt214074-bib-0031] Magge, A. , Setlow, B. , Cowan, A. , and Setlow, P. (2009) Analysis of dye binding by and membrane potential in spores of Bacillus species. J Appl Microbiol 106: 814–824. 19187156 10.1111/j.1365-2672.2008.04048.x PMC2661013

[mbt214074-bib-0032] Marcella, C. , Cui, B. , Kelly, C.R. , Ianiro, G. , Cammarota, G. , and Zhang, F. (2021) Systematic review: the global incidence of faecal microbiota transplantation‐related adverse events from 2000 to 2020. Aliment Pharmacol Ther 53: 33–42. 33159374 10.1111/apt.16148

[mbt214074-bib-0033] Mattila, E. , Uusitalo–Seppälä, R. , Wuorela, M. , Lehtola, L. , Nurmi, H. , Ristikankare, M. , *et al*. (2012) Fecal transplantation, through colonoscopy, is effective therapy for recurrent Clostridium difficile infection. Gastroenterology 142: 490–496. 22155369 10.1053/j.gastro.2011.11.037

[mbt214074-bib-0034] McSweeney, B. , Allegretti, J.R. , Fischer, M. , Xu, H. , Goodman, K.J. , Monaghan, T. , *et al*. (2020) In search of stool donors: a multicenter study of prior knowledge, perceptions, motivators, and deterrents among potential donors for fecal microbiota transplantation. Gut Microbes 11: 51–62. 31122134 10.1080/19490976.2019.1611153 PMC6973337

[mbt214074-bib-0035] Paramsothy, S. , Walsh, A.J. , Borody, T. , Samuel, D. , van den Bogaerde, J. , Leong, R.W. , *et al*. (2015a) Gastroenterologist perceptions of faecal microbiota transplantation. World J Gastroenterol 21: 10907–10914. 26478682 10.3748/wjg.v21.i38.10907 PMC4600592

[mbt214074-bib-0036] Paramsothy, S. , Borody, T.J. , Lin, E. , Finlayson, S. , Walsh, A.J. , Samuel, D. , *et al*. (2015b) Donor recruitment for fecal microbiota transplantation. Inflamm Bowel Dis 21: 1600–1606. 26070003 10.1097/MIB.0000000000000405

[mbt214074-bib-0037] Peng, Z. , Xiang, J. , He, Z. , Zhang, T. , Xu, L. , Cui, B. , *et al*. (2016) Colonic transendoscopic enteral tubing: a novel way of transplanting fecal microbiota. Endosc Int Open 4: E610–613. 27556065 10.1055/s-0042-105205 PMC4993903

[mbt214074-bib-0038] Qi, X. , Li, X. , Zhao, Y.E. , Wu, X. , Chen, F. , Ma, X. , *et al*. (2018) Treating steroid refractory intestinal acute Graft‐vs.‐Host disease with fecal microbiota transplantation: a pilot study. Front Immunol 9: 2195. 30319644 10.3389/fimmu.2018.02195 PMC6167440

[mbt214074-bib-0039] Ren, R.‐R. , Sun, G. , Yang, Y.‐S. , Peng, L.‐H. , Wang, S.‐F. , Shi, X.‐H. , *et al*. (2016) Chinese physicians' perceptions of fecal microbiota transplantation. World J Gastroenterol 22: 4757–4765. 27217707 10.3748/wjg.v22.i19.4757 PMC4870082

[mbt214074-bib-0040] Saha, S. , Mara, K. , Pardi, D.S. , and Khanna, S. (2021) Long‐term safety of fecal microbiota transplantation for recurrent Clostridioides difficile infection. Gastroenterology 160: 1961–1969. 33444573 10.1053/j.gastro.2021.01.010

[mbt214074-bib-0041] See, I. , Iwamoto, M. , Allen‐Bridson, K. , Horan, T. , Magill, S. , and Thompson, N. (2013) Mucosal barrier injury laboratory‐confirmed bloodstream infection: results from a field test of a new National Healthcare Safety Network definition. Infect Control Hosp Epidemiol 34: 769–776. 23838215 10.1086/671281

[mbt214074-bib-0042] Terveer, E.M. , van Beurden, Y.H. , Goorhuis, A. , Seegers, J. , Bauer, M.P. , van Nood, E. , *et al*. (2017) How to: Establish and run a stool bank. Clin Microbiol Infect 23: 924–930. 28529025 10.1016/j.cmi.2017.05.015

[mbt214074-bib-0043] Wang, H. , Cui, B. , Li, Q. , Ding, X. , Li, P. , Zhang, T. , *et al*. (2018) The safety of fecal microbiota transplantation for Crohn's disease: findings from a long‐term study. Adv Ther 35: 1935–1944. 30328062 10.1007/s12325-018-0800-3 PMC6223988

[mbt214074-bib-0044] Wang, J. , Li, X. , Wu, X. , Wang, Z. , Wu, X. , Wang, S. , *et al*. (2021) Fecal microbiota transplantation as an effective treatment for Carbapenem‐Resistant Klebsiella pneumoniae infection in a renal transplant patient. Infect Drug Resist 14: 1805–1811. 34017186 10.2147/IDR.S308308 PMC8131010

[mbt214074-bib-0045] Wang, J.‐W. , Wang, Y.‐K. , Zhang, F. , Su, Y.‐C. , Wang, J.‐Y. , Wu, D.‐C. , and Hsu, W.‐H. (2019) Initial experience of fecal microbiota transplantation in gastrointestinal disease: A case series. Kaohsiung J Med Sci 35: 566–571. 31197926 10.1002/kjm2.12094 PMC11900732

[mbt214074-bib-0046] Wen, Q. , Liu, K.‐J. , Cui, B.‐T. , Li, P. , Wu, X. , Zhong, M. , *et al*. (2020) Impact of cap‐assisted colonoscopy during transendoscopic enteral tubing: a randomized controlled trial. World J Gastroenterol 26: 6098–6110. 33132658 10.3748/wjg.v26.i39.6098 PMC7584059

[mbt214074-bib-0047] Woodworth, M.H. , Carpentieri, C. , Sitchenko, K.L. , and Kraft, C.S. (2017) Challenges in fecal donor selection and screening for fecal microbiota transplantation: a review. Gut Microbes 8: 225–237. 28129018 10.1080/19490976.2017.1286006 PMC5479407

[mbt214074-bib-0048] Wu, X. , Cui, B. , and Zhang, F. (2021) Washed microbiota transplantation for the treatment of recurrent fungal infection in a patient with ulcerative colitis. Chin Med J (Engl) 134: 741–742. 33470649 10.1097/CM9.0000000000001212 PMC7990002

[mbt214074-bib-0049] Wu, X. , Dai, M. , Buch, H. , Bai, J. , Long, W. , Long, C. , *et al*. (2019) The recognition and attitudes of postgraduate medical students toward fecal microbiota transplantation: a questionnaire study. Therap Adv Gastroenterol 12: 1756284819869144. 10.1177/1756284819869144 PMC6724572 31516555

[mbt214074-bib-0050] Xiang, L. , Ding, X. , Li, Q. , Wu, X. , Dai, M. , Long, C. , *et al*. (2020) Efficacy of faecal microbiota transplantation in Crohn's disease: a new target treatment? Microb Biotechnol 13: 760–769. 31958884 10.1111/1751-7915.13536 PMC7111085

[mbt214074-bib-0051] Ye, Z. , Xia, H. , Zhang, R. , Li, L. , Wu, L. , Liu, X. , *et al*. (2020) The Efficacy of Washed Microbiota Transplantation on *Helicobacter pylori* Eradication: A Pilot Study. Gastroenterol Res Pract 2020: 8825189. 33133183 10.1155/2020/8825189 PMC7593733

[mbt214074-bib-0052] Zhang, F. , Cui, B. , He, X. , Nie, Y. , Wu, K. , and Fan, D. (2018) Microbiota transplantation: concept, methodology and strategy for its modernization. Protein Cell 9: 462–473. 29691757 10.1007/s13238-018-0541-8 PMC5960466

[mbt214074-bib-0053] Zhang, F. , Luo, W. , Shi, Y. , Fan, Z. , and Ji, G. (2012) Should we standardize the 1,700‐year‐old fecal microbiota transplantation? Am J Gastroenterol 107 **:** 1755; author reply p.1755–1756. 23160295 10.1038/ajg.2012.251

[mbt214074-bib-0054] Zhang, F. , Wang, H. , Wang, M. , Cui, B. , Fan, Z. , and Ji, G. (2013) Fecal microbiota transplantation for severe enterocolonic fistulizing Crohn's disease. World J Gastroenterol 19: 7213–7216. 24222969 10.3748/wjg.v19.i41.7213 PMC3819561

[mbt214074-bib-0055] Zhang, F. , Wen, Q. , and Cui, B. (2021a) Drainage via colonic transendoscopic enteral tubing increases our confidence in rescuing endoscopy‐associated perforation. Endoscopy 54: E201–E202. 33979865 10.1055/a-1472-5586

[mbt214074-bib-0056] Zhang, T. , Ding, X. , Dai, M. , Zhang, H. , Xiao, F. , He, X. , *et al*. (2021b) Washed microbiota transplantation in patients with respiratory spreading diseases: practice recommendations. Med Microecol 7: 100024. 34046562 10.1016/j.medmic.2020.100024 PMC7547313

[mbt214074-bib-0057] Zhang, T. , Lu, G. , Zhao, Z. , Liu, Y. , Shen, Q. , Li, P. , *et al*. (2020a) Washed microbiota transplantation vs. manual fecal microbiota transplantation: clinical findings, animal studies and in vitro screening. Protein Cell 11: 251–266. 31919742 10.1007/s13238-019-00684-8 PMC7093410

[mbt214074-bib-0058] Zhang, T. , Long, C. , Cui, B. , Buch, H. , Wen, Q. , Li, Q. , *et al*. (2020b) Colonic transendoscopic tube‐delivered enteral therapy (with video): a prospective study. BMC Gastroenterol 20: 135. 32375675 10.1186/s12876-020-01285-0 PMC7203978

[mbt214074-bib-0065] Zhang, F. , Zhang, T. , Zhu, H. , and Borody, T.J. (2019) Evolution of fecal microbiota transplantation in methodology and ethical issues. Current Opinion in Pharmacology 49: 11‐16. 31059962 10.1016/j.coph.2019.04.004

[mbt214074-bib-0059] Zheng, Y.‐M. , Chen, X.‐Y. , Cai, J.‐Y. , Yuan, Y.U. , Xie, W.‐R. , Xu, J.‐T. , *et al*. (2021) Washed microbiota transplantation reduces proton pump inhibitor dependency in nonerosive reflux disease. World J Gastroenterol 27: 513–522. 33642825 10.3748/wjg.v27.i6.513 PMC7896436

[mbt214074-bib-0060] Zhong, H.‐J. , Zeng, H.‐L. , Cai, Y.‐L. , Zhuang, Y.‐P. , Liou, Y.‐L. , Wu, Q. , and He, X.‐X. (2021a) Washed microbiota transplantation lowers blood pressure in patients with hypertension. Front Cell Infect Microbiol 11: 679624. 34458158 10.3389/fcimb.2021.679624 PMC8385408

[mbt214074-bib-0061] Zhong, M. , Buch, H. , Wen, Q. , Long, C. , Cui, B. , and Zhang, F. (2021b) Colonic transendoscopic enteral tubing: route for a novel, safe, and convenient delivery of washed microbiota transplantation in children. Gastroenterol Res Pract 2021: 6676962. 33510783 10.1155/2021/6676962 PMC7826206

[mbt214074-bib-0062] Zipursky, J. , Sidorsky, T. , Freedman, C. , Sidorsky, M. , and Kirkland, K. (2012) Patient attitudes toward the use of fecal microbiota transplantation in the treatment of recurrent Clostridium difficile infection. Clin Infect Dis 55: 1652–1658. 22990849 10.1093/cid/cis809

[mbt214074-bib-0063] Zipursky, J. , Sidorsky, T. , Freedman, C. , Sidorsky, M. , and Kirkland, K. (2014) Physician attitudes toward the use of fecal microbiota transplantation for the treatment of recurrent Clostridium difficile infection. Can J Gastroenterol Hepatol 28: 319–324. 24719899 10.1155/2014/403828 PMC4072236

